# Dual Biologic or Small Molecule Therapy in Pediatric Inflammatory Bowel Disease: A Single Center Experience

**DOI:** 10.3390/children12010075

**Published:** 2025-01-09

**Authors:** Cheng Guo, Jin Zhou, Guoli Wang, Jie Wu

**Affiliations:** Department of Gastroenterology, Beijing Children’s Hospital, Capital Medical University, National Center for Children’s Health, No. 56 Nanlishi Road, Xicheng District, Beijing 100045, China; 498281184@163.com (C.G.);

**Keywords:** Crohn’s disease, dual biologics, inflammatory bowel disease, pediatric, ulcerative colitis

## Abstract

Purpose: Currently, there is no clinical data reported on the therapy of dual biological agents in pediatric-onset inflammatory bowel disease (PIBD) patients in China. The purpose of this study was to evaluate the efficacy and safety of dual biologic therapy or biologics combined with small molecule drugs in refractory PIBD patients in China. Methods: Clinical, laboratory, endoscopic, and ultrasound data of PIBD patients from the Department of Gastroenterology of Beijing Children’s Hospital between January 2021 and October 2024 were retrospectively analyzed. PIBD patients who received dual biologic treatment or a combination of biologic and small molecule therapy were included in this study. Steroid-free clinical remission and adverse events were recorded. Results: In this retrospective study, out of 520 children with IBD, twelve children (2.3%) were diagnosed with refractory PIBD and met the criteria for dual biotherapy, including four with UC (33%) and eight with CD (67%). The median age of patients was 13.64 (range, 1.2–17.1) years at eligibility for dual biologic therapy. There are eight (67%) patients treated with infliximab/ustekinumab (IFX + UST), three (25%) patients with upadacitinib/ustekinumab (UPA + UST), one (8%) patient with infliximab/vedolizumab (IFX + VDZ). At 3, 6, and 12 months of dual biological treatment, 91.2% (11/12), 100% (12/12), and 100% (12/12) patients showed steroid-free clinical remission, respectively. The median fecal calprotectin decreased significantly from 1852.5 µg/g (IQR, 762.5–1988.25) at baseline to 359.0 (IQR, 217.5–730.25) μg/g at 3 months, 113 (IQR, 73.7–256) μg/g at 6 months, and 82.5 (IQR, 40.25–122.25) μg/g at 12 months. Only one CD patient with IFX + UST reported mild elevation of aminotransferase, who recovered after symptomatic treatment. Conclusions: Dual biologic or small molecule therapy may be effective and safe for children with refractory PIBD in China.

## 1. Introduction

Pediatric-onset inflammatory bowel disease (PIBD) is a chronic inflammatory condition of the gastrointestinal (GI) tract, including Crohn’s disease (CD), ulcerative colitis (UC), and unclassified IBD. The incidence rate of PIBD in China has increased in recent years [1,2]. The pathogenesis of IBD involves the environment, genetic predisposition, immunological system, and microbial flora [3,4]. Clinical manifestations are usually more severe in PIBD, especially in very early onset inflammatory bowel disease (VEOIBD), than in adult IBD patients [5].

Anti-TNF therapy may be considered as first-line therapy in pediatric CD with high-risk disease behavior, and as second-line therapy for steroid-refractory UC or patients presenting with acute severe colitis [6]. About 10–40% of PIBD patients are primary nonresponse (PNR) to anti-TNFs [7], and 20–30% of patients are secondary loss of response (SLR) to anti-TNFs [8]. For those PIBD patients who have failed anti-TNF therapy, second-line off-label drugs such as vedolizumab (VDZ) and ustekinumab (UST) are available [9]. However, more than 1/3 of IBD patients failed to achieve steroid-free clinical remission after 12 months of VDZ or UST treatment [6,10,11]. Upadacitinib (UPA) is a small molecule that inhibits the activity of Janus kinase 1 (JAK1), which has been approved for the treatment of moderate-to-severe UC in adults [12], and some studies have shown its effectiveness in refractory PIBD [12,13,14,15]. Some PIBD patients are refractory to any single biological agent treatment and often face repeated emergency visits or surgical treatment. This therapeutic dilemma may be related to the multiple inflammatory pathways involved in the pathogenesis of IBD.

Recently, some researchers have proposed that dual biological agents or the combination of biological agents and small molecule drugs may be an effective therapy for these refractory IBD patients [16,17,18,19,20,21]. A meta-analysis showed that combining biologics or small molecules in the treatment of refractory IBD was promising and appeared to be generally safe with no major side effects [22]. A multicenter study showed that the clinical remission rate of refractory PIBD patients receiving dual biological therapy was 35–63%, suggesting that dual biological therapy may be effective for refractory PIBD [18].

Currently, clinical data reported on dual biological therapy for refractory PIBD are rare. This study aimed to assess the effectiveness and safety of dual biological agents in a PIBD cohort in China.

## 2. Materials and Methods

### 2.1. Data Sources and Recruitment

This is a retrospective study in PIBD with dual biological therapy between January 2021 and October 2024 in our Department of Gastroenterology, Beijing Children’s Hospital. PIBD patients (<18 years) who received combinations of dual biologic agents or biologic and small molecule drug therapy were included in this study. The diagnosis of IBD was based on disease symptoms and endoscopic and histological features [23]. Since UST, VDZ, and UPA are off-label use of drugs for children, an absolute condition for qualifying for treatment was written consent signed by the legal guardian of each patient.

### 2.2. Data Collection

We collected the demographic and clinical data of PIBD patients through the electronic medical records, including age, gender, course of disease, family history, extraintestinal manifestations (EIM), perianal lesions, Paris classification [5], Pediatric Crohn’s Disease Activity Index (PCDAI) [24], Pediatric Ulcerative Colitis Activity Index (PUCAI) [25], Mayo Endoscopy Score for UC [26], Crohn’s Disease Simple Endoscopic Activity Score (SES-CD) [27], erythrocyte sedimentation rate (ESR), C-reactive protein (CRP), fecal calprotectin (FC), and intestinal ultrasound (IUS) examinations. Clinical activity (based on PUCAI/PCDAI score) and laboratory data of patients were collected at baseline, 3 months, 6 months, and 12 months (±4 weeks) after initiation treatment. Endoscopy data was collected for the patients before dual biological therapy, as well as 6 months (±2 months) and 12 months (±2 months) after dual biological therapy. Adverse events were also recorded.

### 2.3. Outcome Assessment

The primary outcome of this study was steroid-free clinical remission (SF-CR) after the initiation of dual therapy, defined as PUCAI or PCDAI ≤ 10, without the use of steroids. Secondary outcome included no steroid clinical response after induction (defined as a reduction of PCDAI/PUCAI by ≥20 points from baseline in the absence of steroids), normal CRP levels (<10 mg/L) and normal FC (<250 μg/g), endoscopic or ultrasound remission, and adverse events. A Mayo endoscopic score of ≤1 and SES-CD < 3 were used as the cut-off values for evaluating endoscopic remission [27,28]. IUS examinations were performed at baseline and postinduction. Ultrasound response is defined as a decrease of ≥25% in bowel wall thickness (BWT) or an absolute decrease of 2 mm in BWT, all in the most severely affected segment. Ultrasound remission is defined as BWT < 3 mm without intestinal wall hyperemia (BWH) [29].

### 2.4. Statistical Analyses

Categorical variables are represented as frequency and percentage (%). Continuous variables are represented as (mean ± standard deviation) or median and interquartile range (IQR, lower quartiles, and upper quartiles). We used the Wilcoxon signed rank test to assess the changes in inflammatory markers (CRP, FC). *p*-values were adjusted by using the Bonferroni correction for multiple comparisons. A two-sided *p*-value < 0.05 was considered statistically significant. SPSS 20.0 (SPSS, Inc., Chicago, IL, USA) was used for statistical analysis.

### 2.5. Ethical Approval

This study was approved by the Medical Ethics Committee of Beijing Children’s Hospital in China (Approval Number: [2024]-E-099-R). Due to the retrospective nature of the study, informed consent was waived.

## 3. Results

### 3.1. Patient Population

In this retrospective study, out of 520 children with IBD, twelve children (2.3%) were diagnosed with refractory PIBD and met the criteria for dual biotherapy, including four with UC (33%) and eight with CD (67%) (Table 1). All twelve patients received dual biological therapy for more than 12 months. The age at diagnosis of PIBD was 12.9 (range, 1.1–15.6) years, and the age at initiation of dual therapy was 13.64 (range, 1.2–17.1) years (see Table 1). Two of the eight CD patients were diagnosed with VEOIBD. One VEOIBD patient was a 1.2-year-old and carried a mutation (c.761_764dup) in the Mediterranean fever (*MEFV*) gene; the other VEOIBD patient was 5.7 years old and carried a duplication of 9p24.3 (chr9: g.50535_323262dup). All patients were identified by gastroenteroscopy for the location and extent of IBD disease. The location and extent of the disease at the initiation of dual therapy for both CD and UC, based on the Paris classification, are shown in Table 1. For CD location, two patients were classified as involving L3 + L4a, four patients were classified as involving L2 + L3, and two patients as involving L2. For CD behavior, two patients were B1 (nonstricturing/nonpenetrating), and the other six patients were B2 (stricturing). In UC patients, four patients were classified as E4 (pancolitis) and S1 (ever severe). Seven children (58%) had EIM, as follows: sacroiliarthritis (three CD), oral ulcers (two CD), pyoderma gangrenosum (one UC), and renal dysfunction (one UC).

### 3.2. Indications and Usage for Dual Biological Therapy

All twelve patients had refractory PIBD. Before starting dual biological therapy, all patients (100%) were previously treated with steroid therapy; the eight CD children were treated with infliximab (IFX) and immunomodulators (azathioprine or tacrolimus), the four UC children were treated with 1–3 biological agents successively (IFX, Adalimumab, VDZ, UST) and 1–2 immunomodulators (azathioprine, tacrolimus). All patients did not achieve clinical remission (PCDAI 30 [IQR, 22.5–43.7] for CD, PUCAI 62.5 [IQR, 33.5–68.5] for UC) (see Table 2). Therefore, the twelve patients were given dual biological treatment. Eight (67%) CD children were treated with IFX + UST, one (8%) UC child was treated with IFX + VDZ; three (25%) UC children were treated with UPA + UST. In addition to dual biological therapy, some patients were treated with azathioprine (two CD patients), exclusive enteral nutrition (EEN) (three CD patients), and methylprednisolone + azathioprine (one UC patient) treatment at the same time (see Table 1).

The interval between the administration of these two biological agents should be at least one week. The induction dose of IFX was 5–10 mg/kg and was given intravenously on day 1, week 2, and week 6. IFX was given intravenously once every 4–8 weeks during the maintenance period. The induction dose of VDZ was 6 mg/kg for patients < 45 kg and 300 mg for patients ≥ 45 kg, and was given intravenously on day 1, week 2, and week 6. VDZ was given intravenously once every 8 weeks during the maintenance period. For UST, the induction dose was 6 to 9 mg/kg for patients weighing < 40 kg, 260 mg for patients weighing 40–55 kg, and 390 mg for patients weighing ≤ 85 kg intravenously; the maintenance dose was 90 mg or 6–9 mg/kg subcutaneously every 8–12 weeks. UPA was orally administered 45 mg per day for 8 weeks, and then the dosage was reduced to 30 mg per day or 15 mg per day as maintenance. Trough levels of biologic therapy preceding and after dual therapy of 12 patients were available (9 IFX, 3 UST) (See Table 3). All applied biologics have reached the optimal dose.

### 3.3. Effectiveness of Dual Biological Therapy

**Figure 1:**
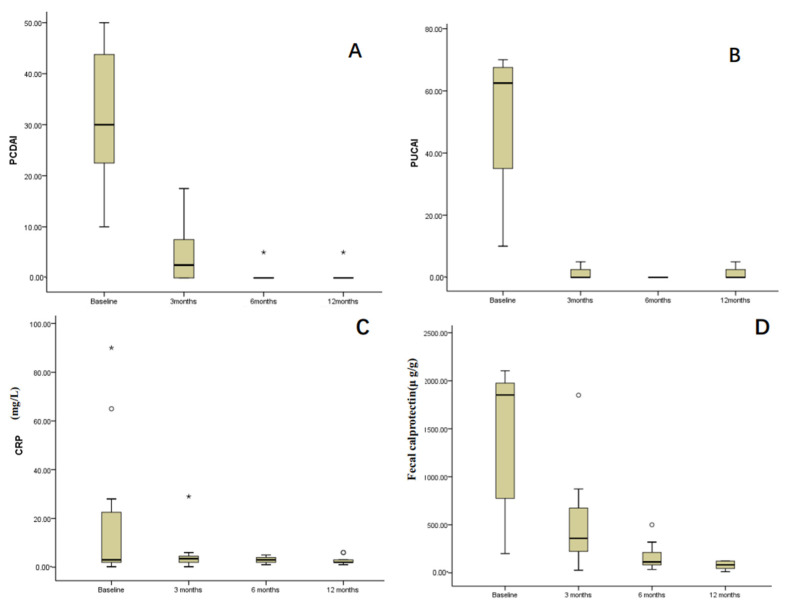
Changes in PCDAI, PUCAI, CRP, and FC at different times before and after dual biological treatment. (**A**) pediatric Crohn’s disease activity index (PCDAI) score, (**B**) pediatric ulcerative colitis activity index score (PUCAI), (**C**) C-reactive protein (CRP), and (**D**) fecal calprotectin (FC). The circle and asterisk are outliers.

Up to the time of data collection in this study, all twelve patients received dual biological therapy for more than 12 months, and no patient discontinued dual biological therapy. After 3, 6, and 12 months of dual biological therapy, SF-CR was observed in 91.2% (11/12), 100% (12/12), and 100% (12/12) of patients, respectively. One patient in the eight CD patients was diagnosed with VEOIBD with an *MEFV* gene mutation (c.761_764dup) and had a steroid-free clinical response after 3 months of dual biological treatment with a PCDAI score of 17.5 and a steroid-free clinical remission after 6 months dual biological treatment with a PCDAI score of 5. The changes in PCDAI/PUCAI scores of the twelve patients at different dual biological treatment times are shown in Figure 1. The median PCDAI in the eight CD patients decreased from 32.5 [IQR, 27.5–42.5] at baseline to 2.5 (IQR, 0–8.75; *p* = 0.003), 0 (IQR, 0–2.5; *p* < 0.001), 0 (IQR, 0–2.5; *p* < 0.001) after 3, 6, and 12 months treatment, respectively. The median PUCAI reduced from 62.5 [IQR, 33.5–68.5] at baseline to 2.5 (IQR, 0–3.75; *p* = 0.013), 0 (IQR, 0–0; *p* < 0.001), 0 (IQR, 0–3.75; *p* < 0.001) after 3, 6, and 12 months treatment after 3 months treatment (Figure 1A,B). All the patients discontinued corticosteroids, immunomodulators, and exclusive enteral nutrition after 3 months of treatment. No child received surgical treatment.

CRP and FC were analyzed at 0, 3, 6, and 12 months of dual biological treatment. At baseline, seven patients had normal CRP; CRP changed from 3 (IQR, 2–25.25) mg/dL at baseline to 3.5 (IQR, 2–4.75) mg/dL at 3 months, 3 (IQR, 2–4) mg/dL at 6 months and 2 (IQR, 2–3) mg/dL at 12 months (Figure 1C), without a statistical difference (*p* > 0.05). CRP in 92% (11/12), 100% (12/12) and 100% (12/12) patients at 3, 6, and 12 months returned to normal, respectively. The level of FC decreased from 1852.5 (IQR, 762.5–1988.25) μg/g at baseline to 359.0 (IQR, 217.5–730.25) μg/g at 3 months, 113 (IQR, 73.7–256) μg/g at 6 months and 82.5 (IQR, 40.25–122.25) μg/g at 12 months (Figure 1D). FC values at 3, 6, and 12 months of dual biological treatment were significantly lower than those at baseline (*p* < 0.001). FC levels in 42% (5/12), 75% (9/12), and 100% (12/12) patients returned to normal at 3, 6, and 12 months, respectively.

After receiving dual biological treatment for 6 months, six patients underwent colonoscopy; all of them achieved endoscopic mucosal remission, including three CD patients with an SES-CD score of 2 and three UC patients with a UC Mayo score of 1. Eight patients achieved ultrasound remission, the other four patients achieved ultrasound response, and the bowel wall thickness decreased more than 2 mm, but the BWT was still >3 mm. All patients (12/12) received dual biological treatment for 12 months; all of them achieved endoscopic mucosal and ultrasound remission.

### 3.4. Adverse Events

A CD patient receiving IFX + UST treatment was found to have elevated serum glutamate pyruvate transaminase levels (70 U/L), which returned to normal after oral administration of compound glycyrrhizin.

## 4. Discussion

To our knowledge, our study is the first retrospective study in China to evaluate the efficacy and safety of dual biologic therapy or a combination of biologics and JAK1 inhibitor therapy in PIBD. In our study, the twelve refractory PIBD patients treated with dual biologics achieved clinical remission and endoscopic healing and maintained remission throughout the follow-up without severe adverse events, which showed promising efficacy and safety in refractory PIBD.

The indications for dual biological agents or biological agents combined with small molecule drug treatment are mainly refractory IBD [30,31,32] and IBD combined with EIM [18,31]. Consistent with previous studies, all patients in this study were refractory PIBD, refractory to various previous therapies (at least one biological agent and immunomodulator). In this study, the blood drug concentration of biologics in children with IBD was normal before the PNR, and the corresponding antibodies did not show a significant increase, suggesting that non-TNF—α/IL-12/IL23 mediated inflammatory pathways may be the main cause of PNR in our study. After the addition of VDZ and UPA, the inflammatory indicators and IBD disease severity scores of the children decreased compared to before dual biologic treatment, which also suggests indirectly verifying the above content.

The age range of PIBD patients receiving dual biological therapy reported in other studies is 3–17 years old [30]. It is unclear whether dual biologics can be used to treat VEOIBD. In contrast to older children, patients with VEO-IBD often have more extensive and severe disease course and are more likely to have a causative monogenic defect. Physicians are increasingly opting for targeted drugs (e.g., IFX, VDZ, UST, and small molecules) rather than traditional empiric VEO-IBD treatments [33]. Reports of dual biologics for the treatment of VEOIBD were rare. Only four patients with VEO-IBD under 6 years old treated with dual biological agents had been reported but had not achieved clinical remission [30]. On the contrary, our study included two patients (1 and 5 years old) with VEO-IBD who were treated with dual biologics (IFX + UST). The treatment was effective, and there were no adverse effects. Our study suggested that dual biological agents may be effective in VEO-IBD patients.

Currently, there are various combination therapies for PIBD, including Adalimumab (ADA)/IFX + VDZ/UST/TOFA (Tofacitinib, TOFA), UST + VDZ, VDZ/UST + TOFA, UPA + UST/VDZ [18,19,30,33,34], and aTNF + VDZ/UST was the most commonly used treatment regimen. TOFA is mainly combined with VDZ, while UPA is mainly combined with UST. A meta-analysis showed that aTNF + UST treatment had the highest clinical response rate for adult IBD (91.6–100%) [22]. The clinical remission rate of PIBD with aTNF + VDZ is 40–61.6%, the clinical remission rate of aTNF + UST for CD is about 67.7–100%, and the clinical remission rate of VDZ + UST is 87.5% [21]. The clinical response rate of TOFA + VDZ/UST was 66.6% [35]. The clinical response rate of UPA + UST was 100% (7/7), and the clinical response rate remained 83% at 6 months [34]. In our study, eight children with CD were treated with IFX + UST, and the steroid-free clinical remission rate was 87.5% at 3 months and 100% at 6 and 12 months. The steroid-free clinical remission rate of IFX + UST/VDZ in our study was higher than that in other studies (86% [30], 67.7% [18]), which may be related to the proactive therapeutic drug monitoring of IFX, timely adjustment of IFX dosage (6.5–9.7 mg/kg) and time interval (q4–8w). We had only 12 patients included in this study. Due to the small sample of patients, the results need to be interpreted with caution. There were three children with UC were treated with UPA + UST, and the steroid-free clinical remission rate was 100% after the treatment of 3, 6, and 12 months, which was consistent with the findings of Spencer et al. [34], who reported 90% (9/10) of patients with UC achieved and sustained SF-CR at 6 months follow-up. Magdalena et al. [30] reported that fecal calprotectin decreased significantly from 1240 µg/g (53–10,100) to 160 µg/g (5–2500; *p* = 0.004) between baseline and Month 4, without a significant decrease in inflammatory markers ESR and CRP. We also only observed a significant decrease in FC in our cohort; no significant changes in CRP were observed. In addition to laboratory examination indicators, endoscopic and ultrasonic indicators were also studied to evaluate the mucosal healing of PIBD patients. Castiglione et al. [36] first proposed that the intestinal wall thickness under IUS < 3 mm was defined as transmural healing. US remission could reflect transmural healing; Spencer et al. [34] reported that 60% (9/15) patients achieved IUS remission at 16 (IQR, 12–20) weeks from UPA start. Our study showed that IUS remission was 66.7% (8/12) and 100 (12/12) after 6 and 12 months of dual biological therapy. Although the remission rate of clinical indicators and endoscopic mucosal healing rate was higher after 6 months of dual biological therapy, the transmural healing rate was lower (66.7%), suggesting that we should integrate laboratory indicators, endoscopic examination, and ultrasound results to evaluate the transmural healing of IBD.

It is unclear when dual biological therapy should be discontinued and switched to a single biologic therapy. Feler [18] reported more than 64.5% (40/62) of patients received dual biological therapy for more than 6–12 months without severe adverse events. Wlazlo et al. [30] reported more than 48% (14/29) of patients received dual biological therapy for more than 12 months without severe adverse events. Our study also showed that all patients (12/12) received dual biological therapy for 12 months without severe adverse events, which might represent a long-term treatment option in some selected patients. In the future, we still need to study further how to gradually discontinue one of the biologics while maintaining the efficacy and safety of children.

There is limited data on the safety of dual biological therapy for PIBD, but overall, it is safe [18,20,22]. The incidence of adverse events [20,22] is about 47–55%, and the incidence of severe adverse events is 12–17%. Adverse events mainly include infections, arthritis, etc. Severe adverse events mainly include severe infections, intestinal obstruction, severe allergic reactions, deep vein thrombosis, etc. Only one case of untreated hyperlipidemia was reported in children with PIBD treated with UPA + UST [34]. In our study, only one child with CD had a mild elevation of transaminase during the therapy of IFX + UST, which returned to normal after symptomatic treatment. No child stopped the combination therapy due to adverse reactions. Due to the small number of participants in this study, further clarification on its safety is needed.

The high cost of dual biological therapy also hinders its application in clinical practice. At present, children who use dual biological agents are all refractory IBD, and the failure of conventional treatment significantly increases the frequency of emergency visits, hospitalizations, and surgical risks, exacerbating their economic burden [37]. Therefore, if the indications and treatment plans for dual biological therapy can be determined early, it may ultimately reduce the treatment costs of patients with refractory IBD.

As one of the largest PIBD medical centers in China, this study is the first and largest single-center study in China to study the efficacy and safety of dual biologics in the treatment of PIBD and the first to mention the treatment of UPA in Chinese refractory UC children. This study has several limitations: (1) Because UST, VDZ. and UPA have not been approved for pediatric IBD in China, the sample size was small, the time after dual biologics therapy was short, and the long-term safety and efficacy cannot be evaluated. Therefore, the efficacy and safety of dual biological therapy should be carefully interpreted. (2) Due to the lack of endoscopic assessment and biomarker detection in some children with economic problems, further assessment of their endoscopic remission cannot be made.

## 5. Conclusions

Our research suggests that dual biological agents or the combination of biological agents and small molecule drugs may be effective and safe in the treatment of refractory PIBD in China. Due to the unique characteristics of the pediatric population, the potential efficacy and the risk of serious adverse events of dual biological therapy should be carefully considered. Future prospective studies with large samples and multiple centers are still needed, focusing on the treatment strategies, long-term effectiveness, and safety of dual biological therapy for PIBD children to benefit more refractory PIBD patients.

## Figures and Tables

**Table 1:** Demographic and clinical characteristics of the study cohort.

Patient	Age at Diagnosis (Years)	Age at Dual Therapy (Years)	Sex	Disease Extent/Location	Disease Behavior	UC Severity	Base Line CRP (mg/dL)	Base Line FC (μg/g)	Base Line Albumin (g/L)	Base Line ESR (mm/h)	Base Line PCDAI/PUCAI	Base Line SESCD Score/Mayo Score	Dual Therapy	EIM	Concomitant Therapy
CD1	15.6	16.2	M	L3L4aG1	B2		2	1950	42.9	2	27.50	16	IFX + UST	oral ulcers	EEN + Azathioprine
CD2	12.6	13.1	F	L2L3G1	B2		1	1830	41.7	5	42.50	24	IFX + UST	sacroiliarthritis	
CD3	5.3	5.7	M	L2L3	B1		0.2	750	44	10	10.00	14	IFX + UST		
CD4	13.4	14.1	F	L2L3	B2		90	2001	40.6	40	32.50	12	IFX + UST		
CD5	13.4	13.8	M	L3L4a	B2		3	294	43.5	51	50.00	14	IFX + UST		EEN
CD6	12.8	17.1	F	L2L3pG1	B2		2	200	47.6	6	45.00	6	IFX + UST	oral ulcers	EEN
CD7	12.8	13.3	M	L2pG1	B2		3	1925	44.8	3	20.00	12	IFX + UST	sacroiliarthritis	Azathioprine
CD8	1.1	1.2	F	L2G1	B1		28	2045	42.3	4	25.00	8	IFX + UST	sacroiliarthritis	Corticosteroids + Azathioprine
UC1	15.5	16	F	E4		S1	16	800	42.8	12	60	3	IFX + VDZ	renal dysfunction	
UC2	10	13	M	E4		S1	2	900	45.5	14	10	3	UPA + UST		
UC3	11.3	13.4	F	E4		S1	17	1875	28.7	14	65	3	UPA + UST	pyoderma gangrenosum	
UC4	14.8	15.1	F	E4		S1	65	2105	24.9	33	70	3	UPA + UST		

Abbreviations: CD, crohn’s disease; UC, ulcerative colitis; M, Male; F, female; L1, involving one-third of the distal ileum only with limited or no cecal disease; L2, colonic involvement only; L3, involvement of both the terminal ileum and colon; L4a, upper disease proximal to the ligament of Treitz; L4b, upper disease distal to the ligament of Treitz and proximal to distal 1/3 ileum; p, perianal involvement; G, growth failure; B1, nonstricturing, nonpenetrating disease; B2, stricturing disease; B3, penetrating disease; E4, pancolic disease; S1, ever severe; EIM, extraintestinal manifestations of IBD; IFX, infliximab; UPA, upadacitinib; VDZ, vedolizumab; UST, ustekinumab; EEN, exclusive enteral nutrition.

**Table 2:** Treatment before the application of dual therapy.

Therapy	All Patients (n = 12)	CD (n = 8)	UC (n = 4)
Exclusive enteral nutrition	9	8	1
Partial enteral nutrition	3	1	2
Corticosteroids	12	8	4
5-ASA	3	0	3
Azathioprine	10	8	2
Tacrolimus	5	1	4
Infliximab	11	8	3
Adalimumab	0	0	0
Vedolizumab	2	0	2
Ustekinumab	3	0	3
Antibiotics	10	6	4
Surgery	1	1	0
Number of biologic agents			
1	9	8	1
2	0	0	0
3	2	0	2
4	0	0	0
Number of immunomodulators			
None	0	0	0
1	9	7	2
2	3	1	2
3	0	0	0
Last therapy before dual therapy			
Biologic agents	0	0	0
Biologic agent and corticosteroids	1	0	1
Biologic agent and IM	10	8	2
Biologic agent, corticosteroids and IM	1	0	1

Abbreviations: 5-ASA, 5-aminosalicylic acid; IM, immunomodulators.

**Table 3:** Trough levels of biological therapy before and after dual therapy.

	Before Dual Therapy				After Dual Therapy			
Patients	IFX Trough Levels (μg/mL)	IFX Antibodies (ng/mL)	UST Trough Levels (μg/mL)	Anti-UST Antibodies (ng/mL)	IFX Trough Levels (μg/mL)	Anti-IFX Antibodies (ng/mL)	UST Trough Levels (μg/mL)	Anti-UST Antibodies (ng/mL)
CD1	13.3	<4			8.7	<4	13.5	0
CD2	13.8	<4			25	<4	14.6	0
CD3	12	<4			6.9	<4	16.5	0
CD4	18	<4			12.2	<4	15.2	0
CD5	16.7	10			8.4	<4	12.1	0
CD6	15	<4			15.1	7	13.4	0
CD7	14	<4			13	<4	16.5	0
CD8	10	<4			14	<4	14.7	0
UC1	12	<4			7.4	<4	15.8	0
UC2			15	0			13.3	0
UC3			16	0			14	0
UC4			15	0			12	0

## Data Availability

The data presented in this study are available on request from the corresponding authors. The data are not publicly available due to privacy reasons.
